# Fascin‐1 as a novel diagnostic marker of triple‐negative breast cancer

**DOI:** 10.1002/cam4.746

**Published:** 2016-05-17

**Authors:** Chao‐Qun Wang, Chih‐Hsin Tang, Hao‐Teng Chang, Xiao‐Ni Li, Yong‐Ming Zhao, Chen‐Ming Su, Gui‐Nv Hu, Tao Zhang, Xin‐Xin Sun, Yue Zeng, Zhang Du, Yan Wang, Bi‐Fei Huang

**Affiliations:** ^1^ Department of PathologyAffiliated Dongyang Hospital of Wenzhou Medical UniversityDongyangZhejiangChina; ^2^ Graduate Institute of Basic Medical ScienceChina Medical UniversityTaichungTaiwan; ^3^ Department of PharmacologySchool of MedicineChina Medical UniversityTaichungTaiwan; ^4^ Department of BiotechnologyCollege of Health ScienceAsia UniversityTaichungTaiwan; ^5^ Hefei National Laboratory for Physical Sciences at Microscale and School of Life SciencesUniversity of Science and Technology of ChinaHefeiAnhuiChina; ^6^ Department of Surgical OncologyAffiliated Dongyang Hospital of Wenzhou Medical UniversityDongyangZhejiangChina; ^7^ Laboratory of BiomedicineAffiliated Dongyang Hospital of Wenzhou Medical UniversityDongyangZhejiangChina; ^8^ Department of Control and Prevention of Endemic & Parasitic DiseasesAnhui Provincial Center for Disease Control and PreventionHefeiAnhuiChina; ^9^ Department of Medical OncologyAffiliated Dongyang Hospital of Wenzhou Medical UniversityDongyangZhejiangChina

**Keywords:** Breast cancer, fascin‐1, immunohistochemistry, triple‐negative breast cancer

## Abstract

In some cases of breast cancer, diagnosis of triple‐negative breast cancer (TNBC) requires further fluorescence in situ hybridization (FISH) for determining human epidermal growth factor receptor 2 (HER2) status. However, few cases undergo FISH in China, leading to difficulty regarding subsequent treatment decisions. Here, we used immunohistochemical analysis to explore expression of fascin‐1, an actin‐bundling protein, as a diagnostic marker of TNBC. A total of 457 cases of breast cancer were divided into four molecular subtypes, including 82 cases (17.9%) of TNBC, 81 (17.7%) of HER2‐enriched, 185 (40.5%) of luminal A, and 109 (23.9%) of luminal B. Positive fascin‐1 expression was seen in 144 cases (31.5%), including 77 (16.8%) strong positive cases. Rates of positive and strong positive expression of fascin‐1 were significantly higher in cases of TNBC than in the other molecular subtypes. In all cases of breast cancer, the sensitivities and specificities of positive and strong positive fascin‐1 expression for predicting TNBC were 87.8% and 80.8%, and 78.0% and 96.5%, respectively. In cases of hormone receptor–negative breast cancer, the sensitivities and specificities of positive and strong positive fascin‐1 expression for predicting TNBC were 87.8% and 61.7%, and 78.0% and 92.6%, respectively. In 24 cases with estrogen receptor (ER)‐, PR‐, and HER2 2 +  equivocal status who underwent FISH, the sensitivity and specificity of strong positive fascin‐1 expression for predicting TNBC were 71.4% and 90.0%. These results suggest that strong positive fascin‐1 expression can be used as a diagnostic marker of TNBC.

## Introduction

Breast cancer is the most common type of cancer and the second leading cause of cancer‐related death among women in the United States 1. Triple‐negative breast cancer (TNBC)—defined as absence of expression of estrogen receptor (ER) and progesterone receptor (PR) as well as no overexpression of human epidermal growth factor receptor 2 (HER2)—accounts for approximately 10% to 20% of all cases of breast cancer 2. TNBC is associated with an aggressive course and poor survival, and unlike patients with ER/PR‐positive or HER2‐overexpressing breast cancer, TNBC is not amenable to hormone therapy or HER2‐targeting therapy, such as trastuzumab 3, 4, 5. Although immunohistochemical (IHC) analysis can be used to assess ER, PR, and HER2 status, some cases of breast cancers are HER2 2+ equivocal. In those cases, fluorescence in situ hybridization (FISH) can be used to further determine HER2 status. However, use of FISH is not universal in China, and the cost is higher than that of IHC analysis; therefore, in some cases of breast cancer, HER2 status remains unclear. In cases of hormone receptor–negative breast cancer, unclear HER2 status leads to uncertain classification as TNBC or HER2‐enriched subtype, resulting in difficulty regarding subsequent targeted therapy decisions.

Fascin‐1, an actin‐bundling protein, is normally expressed in neuronal, mesenchymal, and endothelial cells, and is low or absent in normal epithelial cells 6, 7. Overexpression of fascin‐1 has been reported in several types of carcinoma, including that of the lung 8, colon 9, stomach 10, ovary 11, and breast 12. In a previous study of breast cancer, fascin‐1 expression was associated with TNBC in African American women. That study also provided preliminary analysis of the utility of fascin‐1 expression for predicting TNBC, but the results were not ideal 13. In this study, we used IHC analysis to detect fascin‐1 expression in Chinese women with breast cancer and assessment of staining with new scoring criteria in order to explore the feasibility of fascin‐1 as a novel diagnostic marker of TNBC.

## Materials and Methods

### Patients and tissue samples

Tissue samples were obtained from 457 Chinese women with breast cancer. All specimens were obtained from untreated patients who were undergoing primary surgical treatment at Affiliated Dongyang Hospital of Wenzhou Medical University (Dongyang, Zhejiang, China) from 2007 to 2014. Pathohistologic diagnosis was made according to the breast tumor classification criteria of the World Health Organization 14. Histology grade was based on the Scarff‐Bloom‐Richardson system 15. According to ER, PR, HER2, and Ki‐67 status, all cases of breast cancer were categorized into four subtypes 16: luminal A (ER+ and/or PR+, HER2−, Ki‐67 < 14%), luminal B (ER+ and/or PR+, HER2−, Ki‐67 ≥ 14%; or ER+ and/or PR+, HER2+), HER2‐enriched (ER−, PR−, HER2+), and TNBC (ER−, PR−, HER2−). In cases with ER−, PR−, and HER2 2+ equivocal status, 24 underwent FISH, of whom 10 had HER2 amplification. Thirty‐seven cases with ER−, PR−, and HER2 2+ equivocal status did not undergo FISH and were not included in the study. This study was approved by the Institutional Review Board of Affiliated Dongyang Hospital of Wenzhou Medical University. Samples for diagnostic purposes were taken with prior consent from each patient.

### IHC analysis

Immunohistochemical analysis staining of paraffin‐embedded tissue sections was carried out using the Dako Envision System (Dako, Glostrup, Denmark) following the manufacturer's protocols. Briefly, the sections were submerged in boiling 10 mmol/L sodium citrate (pH, 6.0) for 2 min in a pressure cooker. After being treated with 0.3% hydrogen peroxide for 10 min to block endogenous peroxidase, the sections were incubated with primary antibody for 1 h at room temperature. After washing, the sections were incubated with biotin‐labeled secondary immunoglobulin (Dako) for 40 min at room temperature, followed by incubation with 3,3′‐diaminobenzidine (Dako), also at room temperature. The primary antibodies used were anti‐fascin‐1 mouse monoclonal antibody (clone 55k‐2; diluted at 1:100; Santa Cruz Biotechnology, Santa Cruz, CA), ready‐to‐use anti‐ER rabbit monoclonal antibody (clone SP1, Dako), ready‐to‐use anti‐PR mouse monoclonal antibody (clone PgR636, Dako), HercepTest (Dako), and ready‐to‐use anti‐Ki‐67 mouse monoclonal antibody (clone MIB‐1, Dako).

### FISH analysis

Analysis was performed on formalin‐fixed, paraffin‐embedded tissue sections using commercially available PathVysion HER2 DNA Probe Kit (Abbott‐Vysis, Des Plaines, IL). FISH procedure was developed according to the manufacturer's protocols. The slides were examined using a Leica DM2500 (Leica, Wetzlar, Germany) with appropriate filters for Spectrum Orange, Spectrum Green and the UV filter for the DAPI nuclear counterstain. The signals were recorded with a Leica DFC310 FX CCD camera (Leica, Wetzlar, Germany).

### Assessment of staining

For assessment of staining, the entire tissue section was scanned and scored separately by two pathologists. For assessment of fascin‐1 expression, staining intensity and extent were recorded in cancer cells. Staining intensity was scored as 0 (negative), 1 (weak), 2 (medium), or 3 (strong). Staining extent was scored as 0 (0%), 1 (1%–25%), 2 (26%–50%), 3 (51%–75%), or 4 (76%–100%). Sum of staining intensity and extent scores ≥3 and percentage of invasive tumor cells with unequivocal cytoplasmic staining >5% was considered to be positive for fascin‐1. Sum of scores ≥6 and staining intensity of 3 was considered to be strongly positive for fascin‐1. A case was considered to be ER or PR positive if the percentage of positive invasive cancer cells (nuclear staining) was >5%^12^. HER2 status was assessed according to the 2013 American Society of Clinical Oncology/College of American Pathologists guidelines for HER2 testing in breast cancer 17.

### Statistical analysis

All statistical analyses were carried out using SPSS version 19.0 (SPSS Inc, Chicago, IL). Differences/correlations between groups were compared using Pearson's chi‐squared test for qualitative variables. Utility of fascin‐1 for predicting TNBC was examined by calculating the sensitivity, specificity and positive predictive value (PPV) of its expression. *P* < 0.05 was considered statistically significant.

## Results

### Characteristics and distribution of the study population

Clinical characteristics of the 457 breast cancer patients and distribution of the four molecular subtypes are described in Table 1. There were 82 (17.9%) cases of TNBC, 81 (17.7%) of HER2‐enriched, 185 (40.5%) of luminal A, and 109 (23.9%) of luminal B. In this study population, cases of TNBC had a higher histologic grade and clinical stage compared with the other molecular subtypes (*P *<* *0.01 and 0.05, respectively). No significant difference was observed in age, tumor size, or lymph node metastasis. The distribution of TNBC in this study is consistent with previous research results 3.

**Table 1:** Clinical characteristics of patients and distribution of molecular subtypes

Characteristics	Molecular Subtypes (No. of patients) *n* (%)				*P*‐value
	luminal A (185 cases)*n* (%)	luminal B(109 cases)*n* (%)	HER2‐enriched(81 cases)*n* (%)	TNBC(82 cases)*n* (%)	
Age (years)					
≤35	5 (2.7)	7 (6.4)	4 (4.9)	6 (7.3)	0.171
36–55	117 (63.2)	75 (68.8)	44 (54.3)	52 (63.4)	
>55	63 (34.1)	27 (24.8)	33 (40.8)	24 (29.3)	
Tumor size (cm)					
≤2	112 (60.6)	49 (44.9)	37 (45.7)	35 (42.7)	0.053
2–5	67 (36.2)	55 (50.5)	40 (49.4)	41 (50.0)	
>5	6 (3.2)	5 (4.6)	4 (4.9)	6 (7.3)	
Lymph node metastases					
0	102 (55.1)	59 (54.1)	34 (41.9)	36 (43.9)	0.261
1–3	46 (24.9)	28 (25.7)	22 (27.2)	27 (32.9)	
>3	37 (20.0)	22 (20.2)	25 (30.9)	19 (23.2)	
Tumor grade					
I	20 (10.8)	4 (3.7)	2 (2.5)	2 (2.4%)	0.000
II	155 (83.8)	80 (73.4)	50 (61.7)	29 (35.4)	
III	10 (5.4)	25 (22.9)	29 (35.8)	51 (62.2)	
Tumor stage					
I	71 (38.4)	32 (29.4)	18 (22.2)	18 (21.9)	0.027
II	73 (39.4)	53 (48.6)	36 (44.5)	45 (54.9)	
III	41 (22.2)	24 (22.0)	27 (33.3)	19 (23.2)	
IV	0 (0.0)	0 (0.0)	0 (0.0)	0 (0.0)	

TNBC, triple‐negative breast cancer.

### Association of fascin‐1 expression with molecular classification

**Figure 1:**
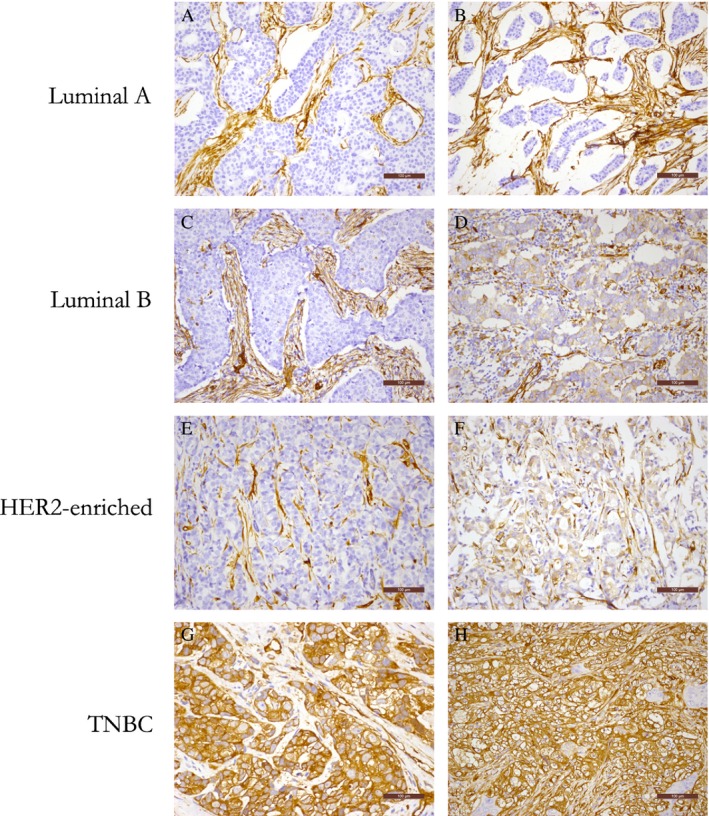
Immunochemical analysis of fascin‐1 expression in four subtypes of breast cancer (200× magnification). **(A, B)** Luminal A subtype. Both cases are negative for fascin‐1 expression in cancer cells. (**C, D)** Luminal B subtype. Case C is negative for fascin‐1 expression in cancer cells, while case D is positive for fascin‐1 expression. **(E, F)** Human epidermal growth factor receptor 2–enriched subtype. Case E is negative for fascin‐1 expression in cancer cells, while case F is positive for fascin‐1 expression. **(G, H)** Triple‐negative breast cancer subtype. Both cases are strongly positive for fascin‐1 expression in cancer cells.

In all cases of breast cancer, the rate of positive fascin‐1 expression was 31.5% (144/457), including 77 (16.8%) strong positive cases. Rates of positive fascin‐1 expression in luminal A, luminal B, HER2‐enriched, and TNBC subtypes were 12.4% (23/185), 16.5% (18/109), 38.3% (31/81), and 87.8% (72/82), while rates of strong positive fascin‐1 expression were 2.2% (4/185), 2.8% (3/109), 7.4% (6/81), and 78.0% (64/82), respectively (Fig. 1). Rates of positive and strong positive expression of fascin‐1 were significantly higher in cases of TNBC than in the other molecular subtypes (both *P *<* *0.01) (Tables 2 and 3).

**Table 2:** Positive fascin‐1 expression in four molecular subtypes

Group	No.	Positive fascin‐1 expression	
		Negative, *n* (%)	Positive, *n* (%)
luminal A	185	162 (87.6)	23 (12.4)
luminal B	109	91 (83.5)	18 (16.5)
HER2‐enriched	81	50 (61.7)	31 (38.3)
TNBC	82	10 (12.2)	72 (87.8)^a^

TNBC, triple‐negative breast cancer.

a *P *<* *0.01.

**Table 3:** Strong positive fascin‐1 expression in four molecular subtypes

Group	No.	Strong positive fascin‐1 expression	
		Negative and weakly positive, (%)	Strong positive, *n* (%)
luminal A	185	181 (97.8)	4 (2.2)
luminal B	109	106 (97.2)	3 (2.8)
HER2‐enriched	81	75 (92.6)	6 (7.4)
TNBC	82	18 (22.0)	64 (78.0)^a^

a *P *<* *0.01.

### Sensitivity, specificity, and PPV of fascin‐1 as a diagnostic marker of TNBC

In all cases of breast cancer, the sensitivity of positive fascin‐1 expression for predicting TNBC was 87.8% (95% confidence interval [CI], 80.7–94.9), while its specificity was 80.8% (95% CI, 76.8–84.8), and the PPV was 50.0% (95% CI, 41.8–58.2). The sensitivity of strong positive fascin‐1 expression for predicting TNBC was 78.0% (95% CI, 69.0–87.0), while its specificity was 96.5% (95% CI, 94.7–98.4), and the PPV was 83.1% (95% CI, 74.7–91.4).

In cases of hormone receptor–negative breast cancer, the sensitivity, specificity, and PPV of positive fascin‐1 expression for predicting TNBC were 87.8% (95% CI, 80.7–94.9), 61.7% (95% CI, 51.1–72.3) and 69.9% (95% CI, 61.0–78.8), respectively. The sensitivity, specificity, and PPV of strong positive fascin‐1 expression for predicting TNBC were 78.0% (95% CI, 69.0–87.0), 92.6% (95% CI, 86.9–98.3), and 91.4% (95% CI, 84.9–98.0), respectively.

In 24 cases of breast cancer with ER−, PR−, and HER2 2+ equivocal status who underwent FISH, 14 cases were diagnosed as TNBC, of whom 10 had strong positive fascin‐1 expression. The others diagnosed as HER2‐enriched had 1 strong positive fascin‐1 expression (Table 4). The sensitivity, specificity, and PPV of strong positive fascin‐1 expression for predicting TNBC were 71.4% (95% CI, 47.7–95.1), 90.0% (95% CI, 71.4–108.6), and 90.9% (95% CI, 73.9–107.9), respectively.

**Table 4:** Strong positive fascin‐1 expression in cases with ER−, PR−, and HER2 2+ equivocal status who underwent FISH

Group	No.	Strong positive fascin‐1 expression	
		Negative and weakly positive, *n* (%)	Strong positive, *n* (%)
FISH (_)	14	4 (28.6)	10 (71.4)
FISH (+)	10	9 (90.0)	1 (10.0)

FISH, fluorescence in situ hybridization.

## Discussion

TNBC accounts for approximately 10–20% of all cases of breast cancer, and is associated with a more aggressive course and a poorer prognosis than other breast cancer subtypes 2, 3. TNBC is different than other breast cancer subtypes with regard to treatment, because it is not amenable to hormone therapy or anti‐HER2‐targeting therapy, and systemic treatment options are limited to cytotoxic chemotherapy 3, 4, 5. Currently, the primary problem associated with diagnosis of TNBC is judgment of HER2 status. Cases with HER2 2+ equivocal results determined by IHC analysis require further detection using FISH. However, few medical centers in China utilize FISH technology, and the cost is higher than that of IHC. In this study, there were 61 cases of breast cancer with ER−, PR−, and HER2 2+ equivocal status; 37 cases did not undergo FISH for the above reasons. Therefore, finding a simpler method for diagnosis of TNBC has important clinical significance regarding subsequent treatment of such patients.

A previous study of breast cancer showed that fascin‐1 expression was associated with TNBC in African American women. That study also provided preliminary analysis of the utility of fascin‐1 for predicting TNBC, but the results were not ideal 13. We think that previous scoring criteria led to low specificity with preliminary analysis of breast cancer, and needed to do further study. In our study, we used two scoring criteria that were different from their criteria to explore the feasibility of fascin‐1 as a novel diagnostic marker of TNBC, including new scoring criteria for strong positive fascin‐1 expression. Our results showed that the sensitivity and specificity of strong positive fascin‐1 expression for predicting TNBC were 78.0% and 96.5% in all cases of breast cancer, 78.0% and 92.6% in hormone receptor–negative cases of breast cancer, and 71.4% and 90.0% in cases of breast cancer with ER−, PR−, and HER2 2+ equivocal status who underwent FISH, respectively. These results indicate that strong positive expression of fascin‐1 can be used as a novel diagnostic marker of TNBC. As mentioned above, among these 61 cases with ER−, PR−, and HER2 2+ equivocal status, 24 cases undergoing FISH determination were sufficient to determine the effectiveness of fascin‐1 used in prediction of the HER2 status of TNBC. Therefore, fascin‐1 could be used as a screening marker of TNBC before utilizing FISH techniques. This finding simplified the process of diagnosis of TNBC by using IHC analysis to preliminarily screen patients, which can relieve the economic burden. Furthermore, to the best of our knowledge, there has been no report of fascin‐1 as a diagnostic marker of other tumors. Therefore, this is a novel finding with important clinical significance.

This study used anti‐fascin‐1 mouse monoclonal antibody (clone 55k‐2) purchased from Santa Cruz Biotechnology. We chose this antibody because many studies have utilized anti‐fascin‐1 antibody with a clone of 55k‐2 from such companies as Santa Cruz Biotechnology 18, Dako 12, and Cell Marque 13. Therefore, we choose this type of fascin‐1 antibody from one of those companies. The cases in this study were all Han Chinese women from the Zhejiang region in China. Therefore, we believe that further multicenter studies in other regions or nations are needed to verify our findings.

## Conflict of Interest

The authors declare no conflicts of interest.
